# Supplementary material for: Exploring multimorbidity clusters in relation to healthcare use and its impact on self-rated health among older people in India
Source: PLOS Glob Public Health. 2023 Dec 28;3(12):e0002330. doi: 10.1371/journal.pgph.0002330 (PMC10754468; doi:10.1371/journal.pgph.0002330)
Supplement: Attachment — Submitted filename: REVIEW REPORT][3rd July, 23.doc [file pgph.0002330.s001.doc]

**Reviewer Report**

**Topic**

Very appropriate and reads fine.

**Abstract**

**Background:** This is lacking in the abstract. The authors should provide a sentence or two on the background.

**Purpose/Aim:** This is apt and reads well.

**Methods:** Appropriate.

**Results:** Adequate.

**Conclusion:** This was adequate.

**Overall comment on the abstract:** Except for the missing background, the abstract was adequate and sufficient.

**Introduction**

**Background:** This was well written and sufficiently addressed the topic under study. However, some minor corrections required:

**Para 2**, sentence 1: Source required.

**Para 3**, sentence 1: Source required.

**Para 4**, sentence 6: “Evidence also suggests reporting poor or good.” This is not very clear. It appears hanging. Either delete or recast.

**Para 4**, last sentence, line 3: “This is also important as this perspective of multimorbidity

clusters and healthcare use provides us with a new dimension of multimorbidity-

related healthcare requirements ***to*** further helps to improve the health status of the

elderly.” Replace “***to***” with “***which***”.

**Problem statement:** Clear and elucidates the problem well.

**Materials and methods**

**Study sample:** Line 9 – “***Separate*** written informed consent was obtained for household and

individual surveys and dried blood spot collection.” Replace “***separate***” with

“***separately***”.

**Chronic health conditions**, page 6, sentence 2: “The list of nineteen self-reported chronic

diseases included hypertension, chronic heart diseases (CHD), chronic obstructive

pulmonary disease (COPD), stroke, chronic bronchitis, asthma, diabetes, cancer or

malignant tumor, arthritis, rheumatism, osteoporosis, mental disorders, thyroid

disease, urinary incontinence, gastrointestinal disorders, skin disease, chronic renal

failure, kidney stones, and high cholesterol.” This sentence reads well, but too

long. Recast into two sentences.

**Statistical analysis:** Adequate.

**Results:** Adequate.

**Discussions**

Adequate and reads well. However, some minor corrections required:

**Page 12**, para 2, sentence 3: “Accumulated evidence indicated that uncontrolled high blood

pressure plays a central role in ***the*** developing cardiovascular (i.e., heart attack,

stroke, diabetes, myocardial infarction, etc.) and non-cardiovascular outcomes (i.e.,

dementia, cancer, oral health conditions, etc.) [50–52].” Please, delete the article

“***the***”.

**Page 13**, para 2, sentence 1: “***While***, we have also provided an important finding on the

linkage between multimorbidity clustering, healthcare use, and SRH.” This sentence

does not read well. Consider a recast.

**Conclusion**

This is adequate and reads well.

**Overall observation**

On the whole, this paper was well written. The authors have demonstrated a good understanding of the subject matter and executed the study pretty well. I congratulate them for achieving this fit.
