# Supplementary material for: Exploring multimorbidity clusters in relation to healthcare use and its impact on self-rated health among older people in India
Source: PLOS Glob Public Health. 2023 Dec 28;3(12):e0002330. doi: 10.1371/journal.pgph.0002330 (PMC10754468; doi:10.1371/journal.pgph.0002330)
Supplement: Attachment — Submitted filename: Response to Reviewers.doc [file pgph.0002330.s002.doc]

**Reviewer Report**

**Reviewer #1**

**Topic**

Very appropriate and reads fine.

**Abstract**

**Background:** This is lacking in the abstract. The authors should provide a sentence or two on the background.

***Response:*** *Thank you for the suggestion. In the revised manuscript, it has been rewritten.*

**Purpose/Aim:** This is apt and reads well.

**Methods:** Appropriate.

**Results:** Adequate.

**Conclusion:** This was adequate.

**Overall comment on the abstract:** Except for the missing background, the abstract was adequate and sufficient.

**Introduction**

**Background:** This was well written and sufficiently addressed the topic under study. However, some minor corrections required:

**1. Para 2**, sentence 1: Source required.

***Response:*** *Comment is incorporated. After the other reviewer’s suggestion, we have rewritten the paragraph and included the source for the same.*

**2. Para 3**, sentence 1: Source required.

***Response:*** *Comment is incorporated.*

**3. Para 4**, sentence 6: “Evidence also suggests reporting poor or good.” This is not very clear. It appears hanging. Either delete or recast.

***Response:*** *Thank you for the observation. In the revised manuscript, it has been rewritten.*

**4. Para 4**, last sentence, line 3: “This is also important as this perspective of multimorbidity clusters and healthcare use provides us with a new dimension of multimorbidity-related healthcare requirements ***to*** further helps to improve the health status of the elderly.” Replace “***to***” with “***which***”.

***Response:*** *Sure****, c****omment is incorporated*.

**5. Problem statement:** Clear and elucidates the problem well.

**Materials and methods**

**6. Study sample:** Line 9 – “***Separate*** written informed consent was obtained for household and individual surveys and dried blood spot collection.” Replace “***separate***” with “***separately***”.

***Response:*** *Thanks for the observation. Comment is incorporated.*

**7. Chronic health conditions**, page 6, sentence 2: “The list of nineteen self-reported chronic diseases included hypertension, chronic heart diseases (CHD), chronic obstructive pulmonary disease (COPD), stroke, chronic bronchitis, asthma, diabetes, cancer or malignant tumor, arthritis, rheumatism, osteoporosis, mental disorders, thyroid disease, urinary incontinence, gastrointestinal disorders, skin disease, chronic renal failure, kidney stones, and high cholesterol.” This sentence reads well, but too long. Recast into two sentences.

***Response:*** *Thanks for the suggestion. We divided it into two sentences in revised manuscript.*

**Statistical analysis:** Adequate.

**Results:** Adequate.

**Discussions**

Adequate and reads well. However, some minor corrections required:

**8. Page 12**, para 2, sentence 3: “Accumulated evidence indicated that uncontrolled high blood pressure plays a central role in ***the*** developing cardiovascular (i.e., heart attack, stroke, diabetes, myocardial infarction, etc.) and non-cardiovascular outcomes (i.e., dementia, cancer, oral health conditions, etc.) [50–52].” Please, delete the article “***the***”.

***Response:*** *Thank you for noticing. It has been deleted in the revised manuscript.*

**9. Page 13**, para 2, sentence 1: “***While***, we have also provided an important finding on the linkage between multimorbidity clustering, healthcare use, and SRH.” This sentence does not read well. Consider a recast.

***Response:*** *Thank you for the suggestion. We have rewritten the paragraph in the revised manuscript..*

**Conclusion**

This is adequate and reads well.

**Overall observation**

On the whole, this paper was well written. The authors have demonstrated a good understanding of the subject matter and executed the study pretty well. I congratulate them for achieving this fit.

Reviewer #2:

1. The introduction section requires substantial improvement. It looks too very descriptive and not enough recent existing studies have been included. I would suggest adding more literatures and add a conceptual framework.

***Response:*** *Thank you for the suggestion. In the revised manuscript, we have revised the introduction section by adding more literatures for enhancing the understanding for the rationale. Also, we included a conceptual framework highlighting the study objectives.*

2. I understand that you have been given a word limit for the abstract, but I think authors should include a sentence informing the reader what the problem is and then move on to the sentence stating the aims of the paper.

***Response:*** *Thank you for noticing, we have revised the abstract with the problem statement along with the specific hypothesis.*

3. The study does not discuss how the findings of this study connect to past research in this field. The authors should revise their Introduction to include references to the relevant literature, particularly newly published works by

Akhtar SN, Saikia N, Muhammad T (2023) Self-rated health among older adults in India: Gender specific findings from National Sample Survey. PLoS ONE 18(4): e0284321. <https://doi.org/10.1371/journal.pone.0284321>

Khan, M.R., Malik, M.A., Akhtar, S.N. et al. Multimorbidity and its associated risk factors among older adults in India. BMC Public Health 22, 746 (2022). <https://doi.org/10.1186/s12889-022-13181-1>

Akhtar, S.N. and Saikia, N. (2022), "Differentials and predictors of hospitalisation among the elderly people in India: evidence from 75th round of National Sample Survey (2017-2018)", Working with Older People, Vol. 26 No. 4, pp. 325-341. <https://doi.org/10.1108/WWOP-11-2021-0055>

Ahmed W, Muhammad T, Maurya C, Akhtar SN (2023) Prevalence and factors associated with undiagnosed and uncontrolled heart disease: A study based on self-reported chronic heart disease and symptom-based angina pectoris among middle-aged and older Indian adults. PLoS ONE 18(6): e0287455. <https://doi.org/10.1371/journal.pone.0287455>

Srivastava, S., Chauhan, S., & Patel, R. (2021). Socio-economic inequalities in the prevalence of poor self-rated health among older adults in India from 2004 to 2014: a decomposition analysis. Ageing International, 46(2), 182-199.

Chauhan, S., Patel, R., & Kumar, S. (2022). Prevalence, factors and inequalities in chronic disease multimorbidity among older adults in India: analysis of cross-sectional data from the nationally representative Longitudinal Aging Study in India (LASI). BMJ open, 12(3), e053953.

***Response:*** *Thank you for sharing these recent articles. We have rewritten the introduction and discussion section in the revised manuscript by adding these literatures wherever it was required.*

4. Please state, how your study is different from the previous existing studies? Justify!

***Response:*** *Sure! Previous studies explored the healthcare utilization in relation to multimorbidity by simply counting the number of diseases, however, these studies did not delve into the various patterns or a cluster of multimorbidity. Understanding healthcare needs in relation to different multimorbidity patterns is crucial for providing patient-centered care and addressing the complexities of treating multiple chronic conditions. In Indian context, only few studies explored the multimorbidity patterns were limited in their examination of associated factors only. By exploring how healthcare use vary across various multimorbidity clusters, healthcare providers can tailor interventions and allocate resources more effectively with more targeted and responsive to their specific needs. Additionally, self-rated health has been explored to understand whether patients with multimorbidity perceive improvements in their health status following healthcare utilization across different multimorbidity clusters. This new assessment will contribute to our understanding of how healthcare interventions impact the well-being of people living with multiple chronic conditions, further emphasizing the importance of patient-centered care.*

5. Why the authors did not use the Chi-squared test? What do you mean by crude models? Do they measure the same thing – association? Why did you use these in the first place? What was the purpose?

***Response:*** *Thank you for the observation. In the revised manuscript, we have created a separate graph for the first column of table 2 where we estimated the prevalence of each cluster while adjusting for age and sex. However, we have replaced this column with the percentage of people utilising healthcare by identified multimorbidity clusters and used chi square test for the same. Furthermore, we used logistic regression for examining the associations by controlling for potential confounding variables. Where, crude model refers to an unadjusted model that includes only the independent variable(s) of interest without considering or adjusting for any potential confounding factors. To obtain more reliable and comprehensive results, we controlled the potential covariates in the third column. Thus, each column holds distinct meaning within its context. I hope this clarifies the concerns.*

6. As can be seen in Results, the authors have selected some variables that aren't mentioned in the section on explanatory variables. All the explanatory factors should be described in detail. The authors may choose to provide a table describing the various classifications and definitions of each explanatory variable. It increases the authenticity and replicability of work.

***Response:*** *Thank you for the noticing and suggestion. We have mentioned the remaining explanatory variables in the revised manuscript. As per your suggestion, we have added a table for the description of the variables.*

7. The mathematical expression of the model used in the study are missing in the methodology section. It is so generic expression. It should include the variables—or at least vectors of variables—for the actual regression in order to increase its value in the paper. As it stands, the generic equation does little beyond the description of variables above.

***Response:*** *Thank you for the suggestion. We have included the mathematical expression of the models according to study variables with more clarity and understanding. I hope it shows the clear understanding.*

8. Why did the author included the socio-demographic distribution, if the author has not shown its association with the outcome variables? Justify.

***Response:*** *Sure!* *In this study, the primary objective was to examine the relationship between multimorbidity clusters and healthcare use and self-rated health. To ensure the validity of the findings, we included sociodemographic variables as covariates. This was done to account for the potential influence of these variables on the associations observed between the multimorbidity clusters and the outcome variable. This approach allows for a more accurate assessment of the independent contribution of the multimorbidity clusters to the outcome variables of interest. Additionally, the covariates were used in calculating the propensity score.*

9. What is the major drawback of this study?

***Response:*** *The major drawback of this study is that it exclusively focused on physical chronic health conditions and did not consider mental health conditions. By not including mental health in the analysis, the study might have missed out on important insights into the interactions between physical and mental health conditions and their combined impact on healthcare utilization and well-being among older individuals. In the revised manuscript, we have mentioned it into the limitation section.*

10. Tables: All tables should have uniformly 2 decimals.

***Response:*** *Thank you for noticing. Comment is incorporated.*

11. the discussion section is underdeveloped. The authors should highlight how the findings of this study contribute uniquely to the body of existing knowledge.

***Response:*** *In the revised manuscript, we have thoroughly discussed the significance of our findings in the context of existing studies.*

12. Any additional policy recommendation that focuses on particular states for the specific issues?

***Response:*** *Sure thing! We have included a paragraph in the revised manuscript highlighting the implication for practice and research.*

13. Please include the source, and captions for each figures properly.

***Response:*** *Comment is incorporated.*

14. There are many grammatical errors. Please revise.

***Response:*** *We have checked and corrected the grammatical errors throughout the revised manuscript.*

15. Please check the writing format of PLOS. Rearrange the headings and subheadings.

***Response:*** *Comment is incorporated.*
