# Supplementary material for: Exploring multimorbidity clusters in relation to healthcare use and its impact on self-rated health among older people in India
Source: PLOS Glob Public Health. 2023 Dec 28;3(12):e0002330. doi: 10.1371/journal.pgph.0002330 (PMC10754468; doi:10.1371/journal.pgph.0002330)
Supplement: Attachment — Submitted filename: Response to Reviewers.docx [file pgph.0002330.s003.docx]

**Reviewer report**

Dear reviewers, thank you for reviewing our revised manuscript and we genuinely appreciate the time and effort you dedicated to this process. In this round of revisions, we have made changes based on your valuable feedback. We believe these alterations have enhanced the manuscript and align it with the expected standards.

**Reviewer #1**: The authors have adequately addressed all issues raised, making the manuscript good enough for publication.

***Response:*** *We sincerely appreciate your time for reviewing our manuscript and your positive feedback. It is reassuring to hear that you find the revisions have adequately addressed the raised issues and that the manuscript is now suitable for publication. Your thorough evaluation and feedback have been invaluable in improving our work. We look forward to the next steps in the publication process.*

**Reviewer #2:** The comments are not fully addressed. The literature section still required substantial revision. Also, the research question is unclear. The paper is very similar to the previous existing studies. There are no new significant findings.

***Response:*** *We appreciate your feedback on our manuscript, and we have taken your comments into careful consideration in the revision process. We understand your concerns about the clarity of the research question and the need for a more substantial revision of the literature section that we have done in the revised manuscript.*

*We want to give you a brief overview for the importance and scope of our study (we explained in the revised manuscript in detail). Previous research in India primarily identified clusters among general population (18 years or above), those aged 15-49 years, and those 45 years or above. Few studies have delved into the specific combinations of chronic NCDs based on population aged 60 years or above. However, they used simply dyad or triad approach, or created dummy variables for identifying the pairs of the diseases. While these methods are relatively simple and straightforward, they are typically limited to studying specific relationships, such as co-occurrence, and may not capture other types of patterns. Only one study by Parul puri et al. used sophisticated statistical approach including Latent Class Analysis (LCA) and network analysis in Indian context but their target population was aged 45 years or above and they were limited to only associated socio-demographic characteristics.*

*Therefore, we used LCA, a complex statistical technique to consider multiple conditions simultaneously provides a more realistic and comprehensive understanding of complex disease patterns, aiding in personalized healthcare, resource allocation, and improved health outcomes. Notably, the identified pattern in our study is distinctive compared to the other studies as we focused on individuals aged 60 years and above, a demographic not extensively explored in this context in India.*

*Secondly, we explored the identified clusters in the context of healthcare use. We found the variations in the magnitude of the ORs for healthcare use, underscoring the complexity of the disease’s occurrence. However, due to limitation of the manuscript length constraints and the availability of the data, we were unable to explore specific healthcare use like inpatient and outpatient care, polypharmacy, numbers of days spent in the hospital inpatient, healthcare expenditure and medication adherence. These avenues remain open for future exploration, as indicated by adding section “Implication for practice and research” in the revised manuscript.*

*Thirdly, we addressed the crucial and considerable aspect of subjective health status following healthcare use among individuals with different identified disease clusters. As highlighted in the literature review, this is essential for improving healthcare quality and understanding patient complexity. A notable finding in our study is that subjective health does not improve even after healthcare use, which represents a novel and significant contribution. However, it's important to note that the cross-sectional nature of the data is a limitation, but it provides valuable insights for other researchers and underscores the need for further longitudinal investigations.*
